# Co-Hydrothermal Carbonization of Goose Feather and Pine Sawdust: A Promising Strategy for Disposal of Sports Waste and the Robust Improvement of the Supercapacitor Characteristics of Pyrolytic Nanoporous Carbon

**DOI:** 10.3390/molecules30010026

**Published:** 2024-12-25

**Authors:** Tingyu Ma, Jieni Wang, Xiaobo Han, Chuanbing Zhang, Yahui Xu, Leichang Cao, Shuguang Zhao, Jinglai Zhang, Shicheng Zhang

**Affiliations:** 1 Henan Key Laboratory of Protection and Safety Energy Storage for Light Metal Materials, College of Chemistry and Molecular Sciences, Henan University, Kaifeng 475004, China; 17539120574@163.com (T.M.); jieniwang@henu.edu.cn (J.W.); 2138030016@henu.edu.cn (X.H.); zhangjinglai@henu.edu.cn (J.Z.); 2 School of Physical Education and Sport, Henan University, Kaifeng 475004, China; 3 Huaxia Besince Environmental Technology Co., Ltd., Zhengzhou 450018, China; zhangchuanbing@besince.cn (C.Z.); xuyahui@besince.cn (Y.X.); 4 Shanghai Key Laboratory of Atmospheric Particle Pollution and Prevention (LAP3), Department of Environmental Science and Engineering, Fudan University, Shanghai 200433, China; zhangsc@fudan.edu.cn

**Keywords:** sports waste, supercapacitor, co-hydrothermal carbonization, capacitive performance

## Abstract

Discarded sports waste faces bottlenecks in application due to inadequate disposal measures, and there is often a neglect of enhancing resource utilization efficiency and minimizing environmental impact. In this study, nanoporous biochar was prepared through co-hydrothermal carbonization (co-HTC) and pyrolytic activation by using mixed goose feathers and heavy-metals-contaminated pine sawdust. Comprehensive characterization demonstrated that the prepared M-3-25 (Biochar derived from mixed feedstocks (25 mg/g Cu in pine sawdust) at 700 °C with activator ratios of 3) possesses a high specific surface area 2501.08 m^2^ g^−1^ and abundant heteroatomic (N, O, and Cu), exhibiting an outstanding physicochemical structure and ultrahigh electrochemical performance. Compared to nanocarbon from a single feedstock, M-3-25 showed an ultrahigh capacitance of 587.14 F g^−1^ at 1 A g^−1^, high energy density of 42.16 Wh kg^−1^, and only 8.61% capacitance loss after enduring 10,000 cycles at a current density of 10 A g^−1^, positioning M-3-25 at the forefront of previously known biomass-derived nanoporous carbon supercapacitors. This research not only introduces a promising countermeasure for the disposal of sports waste but also provides superior biochar electrode materials with robust supercapacitor characteristics.

## 1. Introduction

With the swift advancements in global science and technology coupled with the accelerated pace of industrialization, a substantial quantity of sports and daily necessities have been manufactured to cater to the demands of individuals for sports training and for work or day-to-day life [1,2,3]. However, the lack of proper measures for effective resource utilization has led to waste of recyclable resources, the occupation and pollution of land resources, and the destruction of ecological balance [4,5,6]. The recently concluded Paris Olympic Games garnered extensive attention towards solid waste management and associated environmental challenges [7]. The Olympic Games leverage a significant amount of infrastructure that utilizes waste plastic, cardboard, wood, and other materials as environmental protection measures, largely reducing carbon emissions and minimizing environmental pollution [8]. Globally, 2.01 billion tons of waste is generated annually and by 2050 this is estimated to hit 3.4 billion tons. Lignocellulosic biomass comprises almost 63% of the total waste mass [9]. Materials with high resource recovery value derived from used sporting goods waste and infrastructures, such as badminton feathers, wood, pine sawdust, etc., are still not effectively recycled. Due to the high global demand for wood and the widespread distribution of the wood processing industry, the amount of discarded pine sawdust is considerable [10,11]. Failure to devise an effective strategy for managing this sporting goods waste not only undermines the objective of green and sustainable development but also constitutes an additional waste of resources and energy.

Since the industrial revolution, the excessive consumption of fossil fuels, including coal, oil, and natural gas, has triggered global environmental problems, encompassing an energy crisis and global warming, which has stimulated the exploration of new alternative energy sources [12,13,14]. Supercapacitors (SCs) with fast charging speed, long cycle life, high energy density and power density, and geared towards green environmental protection, provide a promising method for the rapid storage of renewable energy and show a wide range of applications in new energy power generation systems, consumer electronics, automotive transportation and other fields [15,16,17]. Feathers from poultry used in badminton, such as goose feathers, chicken feathers, duck feathers, etc., have an extremely high nitrogen content [18]. Doping them into biochar as an exogenous nitrogen element can not only omit the chemical nitrogen doping steps, but also increase the content of heteroatoms in biochar, enrich its pore structure and surface active sites, and improve its stability [19,20,21,22]. How to utilize discarded sports equipment, specifically badminton shuttlecocks, as a potential recyclable resource and to compound it as an auxiliary material into the preparation process for biochar, proposing reasonable resource utilization measures, and producing excellent nitrogen-rich electrode materials, is a project full of hope and challenge.

Based on the need to improve the performance of supercapacitors, the doping of nitrogen, oxygen, and other heteroatoms in biochar is regarded as an effective method to effectively enhance the performance of carbon-based supercapacitors [23,24]. For example, Geng et al. [25] used *Spirulina platensis* as the carbon and nitrogen source to prepare porous carbon, which showed abundant heteroatomic oxygen (13.78%) and nitrogen (2.55%) and had an exceptional capacitance of 348 F g^−1^. Yu et al. [26] synthesized P-doped hierarchical porous carbon by using *Typha orientalis* leaves with high phosphorus content, possessing a large specific surface area (SSA) of 1975 m^2^ g^−1^ and a high specific capacitance of 324 F g^−1^ provided by P self-doping. Samage et al. [27] researched N, O, and Fe self-doped electro-active carbon material for zinc-ion hybrid capacitor derived Solanum melongena, which delivered an excellent specific capacitance of 313.08 F/g. However, most reports have only concentrated on how to dope more heteroatoms to enhance the electrochemical performance of supercapacitors, ignoring the importance of circular stability and sustainability and not proposing a promising approach for addressing the large amount of solid waste with potential for recycling. Although the doping of elements such as nitrogen and oxygen can induce pseudo-capacitance effects, it simultaneously introduces irreversible redox reactions [28,29]. While these reactions may initially elevate the total specific capacitance by augmenting pseudo-capacitance, their irreversible nature leads to the gradual diminution of pseudo-capacitance over numerous constant current charge–discharge cycles, ultimately resulting in capacitance degradation [30,31]. Therefore, apart from pursuing the high specific capacitance of biochar, how to endow it with circular stability, sustainability, and as an effective measure to deal with the recycling potential of sports solid waste is also worth noting. 

To fill this gap, we propose a promising strategy for sports waste disposal by using co-hydrothermal carbonization, simultaneously resolving the problem of the issue of sports waste and enhancing the electrochemical performance of supercapacitors. Nanoporous biochar was prepared by incorporating discarded badminton feathers as auxiliary materials into the hydrothermal carbonization process of pine sawdust containing heavy metals. To gain a deeper understanding of the effects of the raw material ratio and reaction conditions, morphological characteristics, pore size distribution, elemental analysis, and phase analysis of the as-synthesized hydro-char and porous carbon materials were investigated. Furthermore, the electrochemical properties of the samples were also explored. In addition to providing a promising countermeasure against the resource waste from sports, this study also helps to expand our current understanding of the co-HTC of different forms of biomass to more easily prepare high-performance electrochemical energy storage materials.

## 2. Results and Discussion

### 2.1. Physical and Chemical Characterization of Nanoporous Activated Biochar Material

**Figure 1:**
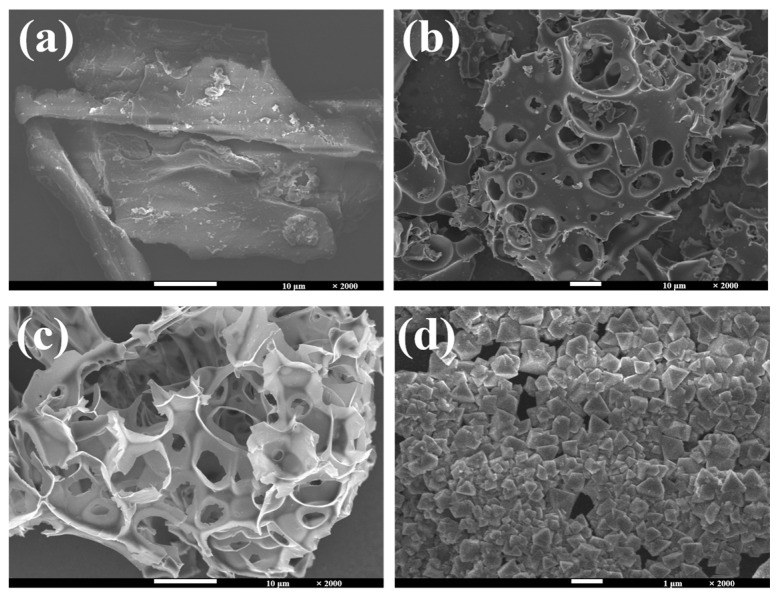
SEM images of (**a**) M-25, (**b**) M-2-25, (**c**) M-3-25, (**d**) M-3-50.

In order to investigate the morphological structure of the prepared carbon materials, the surface morphological structure of the most representative nanoporous carbon and hydro-char samples was observed by SEM, revealing the influence of raw material ratio and reaction conditions on the surface texture of carbon materials (Figure 1). In Figure 1a, the inactivated hydro-char M-25 shows a pyknotic structure with a rough surface and no pore size, which affects its application in the field of electrochemistry. Activated carbon material M-2-25 (Figure 1b) has a porous structure with a variety of pore shapes, but the distribution of these pores is not uniform enough, indicating that the degree of activation is not complete. At a higher activation ratio, a more developed pore structure of M-3-25 was observed (Figure 1c). This pore structure was more evenly distributed on the “honeycomb-like” porous carbon, compared to other samples. The developed pore structure and the addition of heavy metal elements can adjust the electrochemical activity and specific surface area of the electrode material, optimizing the electrochemical performance [32,33]. Energy dispersive spectral analysis (EDS) was carried out on M-3-25 (Figure S1, Supplementary Materials). The results of element spectra showed that the distribution of C, N, O, and Cu elements was uniform, indicating that the nitrogen and heavy metal elements were successfully doped into the nanoporous biochar in the process of sample preparation. With the increase of Cu content in the sample, there are obvious granular sediments in some pores of M-3-50, which occupy the pore structure of nanoporous carbon (Figure 1d). A clogged pore can lead to a decrease in specific surface area and active sites, the blockage of ion transport, and a decrease in electrochemical stability. The M-4-25 (Figure S2) showed a collapsed pore structure, the relatively poor pore structure indicating that too high an activation ratio is not conducive to the development of the pore structure. Compared with pine sawdust not compounded with goose feather, the sample with moderate heavy metal content after composite (M-3-25) has the best morphology and best developed pore structure, which has great potential for electrochemical application.

**Figure 2:**
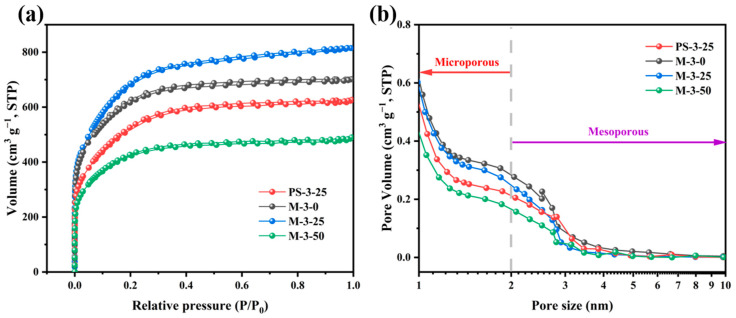
(**a**) N_2_ adsorption and desorption isotherms of porous carbons. (**b**) Pore size distribution diagram of porous carbons.

The specific surface area and pore structure distribution of activated carbon samples were analyzed by the N_2_ absorption–desorption method. All samples showed a combination of types I and IV isotherms (Figure 2a), and hysteresis loops were relatively insignificant, possibly because pore size distribution in the material was relatively uniform and microporous [34,35], which is consistent with the results in Table 1. At low relative pressure (P/P_0_ < 0.05), the isotherms exhibited a sharp increase in adsorption capacity, indicating the presence of abundant micropores. However, at high relative pressure (P/P_0_ > 0.4), the adsorption and desorption processes are not completely reversible, and there is a weak hysteresis effect, which is shown as an H4 hysteresis loop with a certain mesoporous structure. All the samples had abundant hierarchical microporous (under 2 nm) and mesoporous (2–50 nm) structures (Figure 2b). The high specific surface microporous area increases the contact area between the electrode and the electrolyte, improving the charge transport and the electrochemical reaction rate. At the same time, the mesopore provides enough space for the ion transport in the material, which means that the prepared carbon material can be used as an excellent ion transport channel and improve electrochemical performance [36,37]. The rise in specific surface area of PS-3-0 and M-3-0 (1327.36 and 2309.43 m^2^/g) is due to feather biomass, as an additional auxiliary material, having a high protein and nitrogen content. During pyrolysis, these elements form volatile nitrogen compounds that deposit in pores, enhancing pore size distribution and complexity [38]. For M-2-25 and M-4-25, the increase in the proportion of activators enables more KOH to participate in the pyrolysis reaction and produce more H_2_, CO_2_, etc., which contributes to the generation of micropores and mesopores. Nevertheless, at excessive KOH, the carbon skeleton of M-4-25 may be destroyed by KOH erosion, and the pore structure collapses to form a blockage, which cannot effectively improve electrochemical performance. In addition, we found that, with the increase of Cu content in the sample, the specific surface of M-3-0, M-3-25, and M-3-50 continued to decline, (2309.43, 25.1.08, 1555.15 m^2^ g^−1^). This is because, when the dose of copper is small, the uniform distribution of copper particles has little influence on the pore channels. At the same time, the catalytic action of copper particles may promote the graphitization of carbon materials, and at the same time produce gases, leaving micropores in the carbon materials. When the dose of copper increases, more copper particles block the pore structure on the surface of the carbon material, resulting in a decrease in the specific surface area [39,40]. Overall, the activated carbon materials have a high specific surface area and abundant pore structure. Activation ratio and heavy metal content can affect the structural properties of nanoporous carbon, and then affect its electrochemical performance. The performance of the activated carbon electrode material was well regulated by adjusting the two reaction conditions.

**Figure 3:**
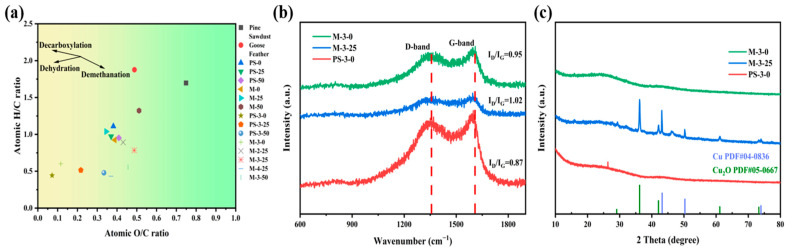
(**a**) Van Krevelen diagram of feedstocks, hydro-chars, and porous carbons, (**b**) Raman patterns of porous carbons, (**c**) XRD spectra of porous carbons.

The elemental composition and yield of hydro-char and activated carbon obtained from the raw materials after hydrothermal conversion and pyrolytic activation are shown in Table 2. Figure 3a shows the Van Krevelen diagram. After HTC, the O/C of Pine Sawdust decreased from 0.75 to 0.38, indicating that dehydration and decarboxylation reactions occurred during HTC [41]. The H/C of hydro-chars showed a declining trend with the addition of goose feather in co-HTC, while the O/C showed a fluctuating trend. In the subsequent process of high temperature pyrolysis activation, hydrogen and oxygen elements in biological carbon are gradually removed, resulting in a decrease in the ratio of H/C and O/C in activated carbon, and an increase in the degree of aromatization. The improvement in the degree of aromatization of biochar makes the carbon atoms in biochar more closely arranged, forming a more perfect conductive network, stronger electron supply capacity, and higher chemical stability, which helps to improve the electrochemical performance of carbon materials [42,43,44].

Figure 3b shows the Raman spectrum of the samples. The peaks around 1367 and 1605 cm^−1^ correspond to the D and G bands, respectively. The D band corresponds to the disordered carbon structure, while the G band is related to the graphitic carbon of the as-prepared sample. The intensity ratio of D to G peaks (I_D_/I_G_) of the M-3-0 and PS-3-0 are 0.95 and 0.87, indicating that the Co-HTC of feathers with high nitrogen content contributes to the increase of disordered carbon [45]. The I_D_/I_G_ ratios of M-3-0 and M-3-25 were 0.95 and 1.02, respectively, showing that a moderate amount of Cu can increase the graphitization degree of the samples, thus enhancing the specific surface area and electrochemical performance of the electrode material.

The crystallinity and phase purity of the prepared electrode material were analyzed by powder X-ray diffraction (XRD). The peaks in Figure 3c show the XRD patterns of the samples, in which the peaks of Cu_2_O (PDF#05-0667) and Cu (PDF#04-0836) are associated with the cubic phase structure [46]. By comparing the XRD pattern of the M-3-25 with the above standard PDF card, the peak is found at 2θ values 43.3° and 50.5°, attributed to the (111) and (200) planes, respectively, which proves the formation of Cu. Similarly, the peak at 2θ values 36.5° and 42.1° are attributed to the (111) plane, demonstrating the formation of Cu_2_O. Meanwhile, for M-3-0 and PS-3-0, no heavy metals were detected. The wide peaks in the 2θ range of 15–25° are correlated with the nanoporous structure of the material.

**Figure 4:**
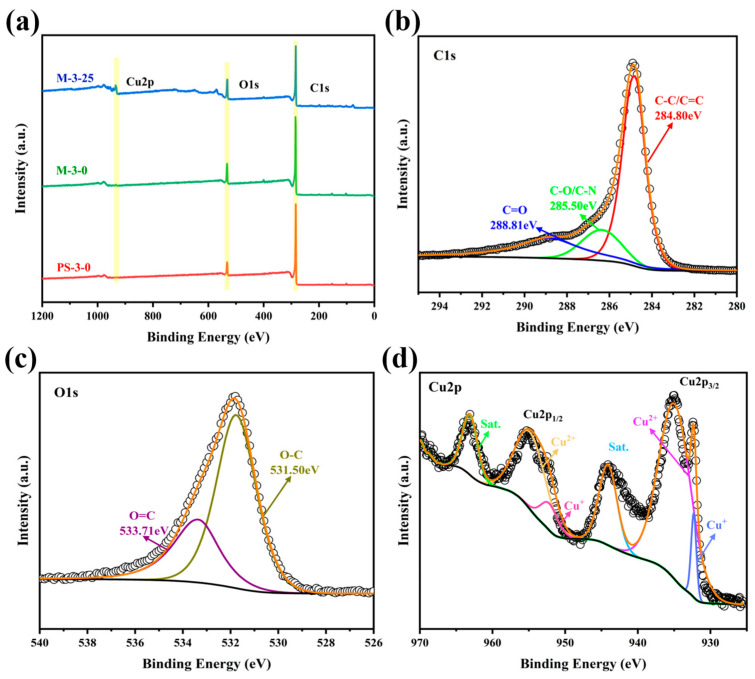
(**a**) Full-scan XPS measurement spectra of samples. (**b**–**d**) Fine spectra of C 1s, O 1s, and Cu2p of M-3-25.

The chemical and electronic states, and elemental composition, of nanoporous biochar samples M-3-25, M-3-0, and PS-3-0 were analyzed by X-ray photoelectron spectroscopy (XPS). The full-scan XPS spectra in Figure 4a revealed that M-3-25 contains Cu (Cu 2p), O (O 1s), C (1s), etc. Deconvoluting the high-resolution spectra was performed by Gaussian fitting method. Figure 4b shows the existence of deoxygenated carbon (C-C) at 284.80 eV, oxygenated carbon (C-O, 285.50 eV), and (C=O, 288.81 eV) [46,47]. The O1s signal of M-3-25 is shown in Figure 4c, and the O 1s spectra are deconvoluted into two oxygen-based components: O-C (531.50 eV) and O=C (533.71 eV) [48,49]. The oxygen-containing groups increase the force between biochar and aqueous electrolytes and improve the wettability and interfacial stability of carbon materials [50,51]. Figure 4d shows the high-resolution Cu 2p spectrum of M-3-25. The two peaks at binding energies of 933.97 and 952.97 eV are ascribed to Cu^+^ of Cu_2_O. Similarly, the other two peaks at binding energies of 935.51 and 955.17 eV are ascribed to Cu^2+^ of CuO [47,52]. Moreover, the noticeable satellite peaks at 944.71 eV are related to CuO, and the peaks at 963.12 eV are related to Cu_2_O [53]. The XPS analysis of samples further supports the XRD results and confirm the successful synthesis of incorporated nanoporous biochar electrode materials.

### 2.2. Electrochemical Performance of Three Electrodes

**Figure 5:**
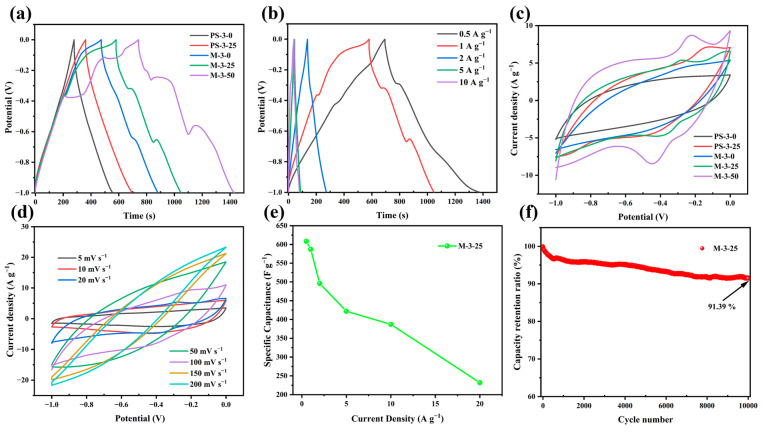
Electrochemical performance of samples in the three-electrode system. (**a**) GCD curves, (**b**) GCD curves of M-3-25 at different densities from 0.5 to 20 A g^−1^, (**c**) CV curves, (**d**) CV curves of M-3-25 at different scanning rates from 5 to 200 mV s^−1^, (**e**) specific capacitance of M-3-25 at different current densities, (**f**) electrochemical cycle test of M-3-25 at 10 A g^−1^.

**Figure 6:**
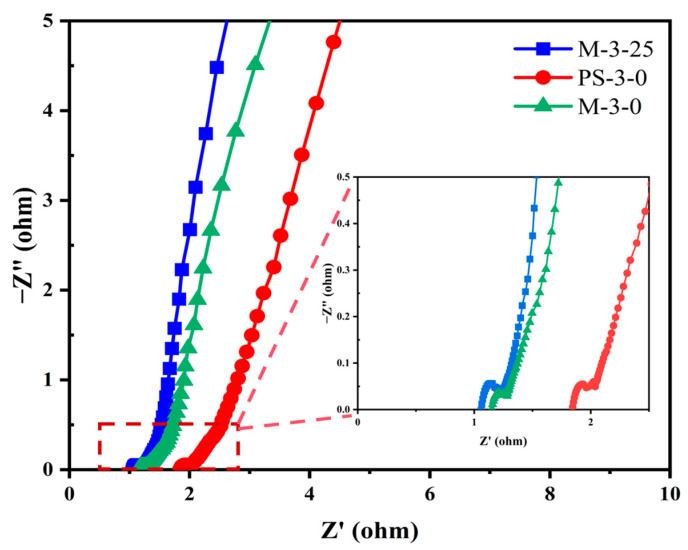
Nyquist plots of M-3-25, PS-3-0, and M-3-0.

To explore the charge storage behavior of the prepared samples in supercapacitors, cyclic voltammetry (CV), galvanostatic charge–discharge (GCD) tests, and electrochemical impedance spectroscopy (EIS) were first performed on a three-electrode system and a two-electrode system in a 6M KOH electrolyte solution. The GCD curves of five samples at a current density of 1 A g^−1^ are presented in Figure 5a. All GCD curves exhibited an almost isosceles triangular shape, indicating excellent charge–discharge characteristics and electrochemical reversibility. For samples containing heavy metals, we found that the GCD curve would bend slightly during the charge and discharge process, especially on the samples of M-3-25 and M-3-50, while this was not the case for other samples. This is because the presence of heavy metals may change the microstructure of electrode materials, such as specific surface area and pore structure, and participate in the electrochemical reaction as an active site, thus affecting the efficiency and path of the electrochemical reaction, resulting in the bending of the GCD curve [54]. Figure 5b shows the GCD curves of M-3-25 at different current densities. All GCD curves maintained relative symmetry, revealing great electrochemical reversibility, the stability of electrode materials, and the effective transfer of ions at the electrode–electrolyte interface. At the scan rate of 20 mV s ^−1^, different samples showed different CV curve shapes Figure 5c. Notably, M-3-50 exhibited the largest CV integral area and the longest charge–discharge time (Figure 5a,c), confirming the highest charge storage capacity. However, elevated heavy metal content in the M-3-50 altered its carbon structure, changing parameters like grain size, surface area, and pore size, resulting in the distortion of the GCD curve, leading to excessive fluctuations and unstable charge–discharge cycles. To sum up, M-3-25 exhibited less fluctuation, more stability, and better overall performance in the electrochemistry test, possessing excellent asymmetric rectangular shapes, and indicating a fast electrochemical reaction and the behavior of dominant EDLCs. The CV curves of M-3-25 at scan rates (5–200 mV s^−1^) are shown in Figure 5d. The CV curve maintained approximately a rectangular shape at low scan rates (5–10 mV s^−1^), indicating its quick ion transport capability. With the increase in scanning rate, the CV curve shows a tendency to distort because, at a high scanning rate, ion diffusion is limited, resulting in uneven electrode surface charge and CV curve deformation [55,56]. The specific capacitance at different current densities (0.5–20 A g^−1^) was calculated according to the discharge curve and equation (1). Figure 5e shows the specific capacitance of M-3-25 at different current densities. Apparently, M-3-25 exhibits ultrahigh specific capacitance (581.74 F g^−1^) at a current density of 1 A g^−1^, indicating its phenomenal charge storage capacity. The cyclic stability of M-3-25 was tested at 10 A g^−1^ (Figure 5f). The capacitance of M-3-25 could still be maintained at 91.39% after 10,000 cycles, demonstrating its excellent cyclic performance. EIS measurements of three samples were studied to evaluate electrochemical performance. The equivalent circuit diagram is shown in Figure S3, where R_s_ denotes the equivalent series and R_ct_ represents the charge transfer resistances. Notably, within the low-frequency spectrum, a trendline with an inclination was observed, which corresponds to the Warburg impedance (W). The Warburg impedance is intimately linked to the diffusion coefficient of the charge-carrying substance within the solution [57]. The Nyquist plots in Figure 6 show approximately vertical lines in the low-frequency region, revealing their excellent EDLC behavior [58]. Specifically, M-3-25 exhibited significantly reduced R_s_ (0.805 U) and R_ct_ (0.077 U), indicative of its low internal resistance and superior conductivity. This diminished internal resistance further minimized thermal losses, potentially extending the operational lifespan of M-3-25.

### 2.3. Electrochemical Performance of Two Electrodes

**Figure 7:**
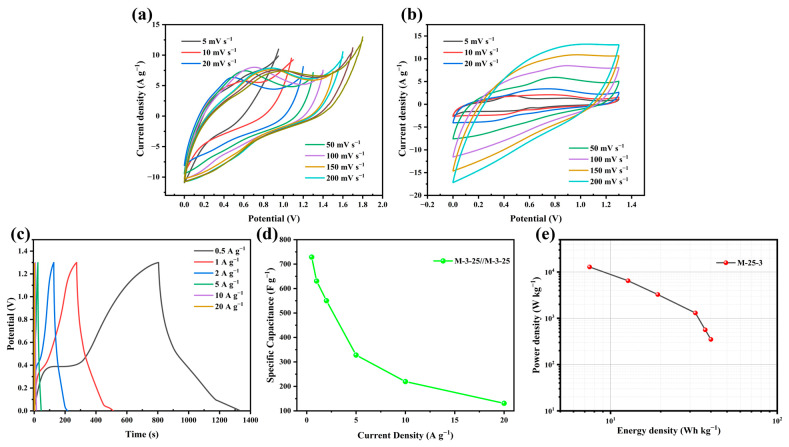
(**a**) CV curves of M-3-25 at different open circuit voltages, (**b**) CV curves of M-3-25 at 1.3 V at open-circuit voltage, (**c**) GCD curves of M-3-25 at different current densities, (**d**) specific capacitance of M-3-25//M-3-25 at different current densities, (**e**) relationship between energy density and power density of M-3-25.

To further evaluate the performance of M-3-25, we assembled a symmetric supercapacitor M-3-25//M-3-25 using 6 M KOH as the electrolyte. The CV test was performed in the voltage window of 0-1.8 V at a scan rate of 20 mV s^−1^ (Figure 7a) to test the rate performance of the symmetric supercapacitor. The emergence of the anode current in the CV curve upon increasing the voltage window to 1.3 V suggested the stable operation of the symmetric supercapacitor within the voltage range of 0–1.3 V. As depicted in Figure 7b, the CV curves exhibited a well-maintained rectangular shape across varying scan rates, thereby validating the superior capacitive properties of prepared nanoporous carbon materials [56]. Additionally, the GCD curves and the specific capacitance at different current densities (Figure 7c,d and Equation (2)) revealed an isosceles triangular profile at different current densities, indicative of the symmetric supercapacitor’s excellent rate capability and rapid reversibility during charging and discharging. Notably, the specific capacitance of the symmetric supercapacitor attained a peak value of 630.6 F g^−1^ at a current density of 1 A g^−1^, underscoring its remarkable charge storage capability. To assess the comprehensive performance of the M-3-25//M-3-25 configuration, we derived the relationship between energy density and power density from the GCD curves, through Equations (3) and (4), respectively, as illustrated in Figure 7e. It is worth noting that the energy density achieved a peak value of 42.16 W h kg^−1^ at a power density of 398.17 W kg^−1^, and it maintained a commendable value of 12.75 W h kg^−1^ even at a significantly higher power density of 6463.30 W kg^−1^. Overall, the two-electrode system of the M-3-25 possesses high capacity, excellent cycle stability, and superior rate performance. Table 3 lists the performances of some recently reported biomass electrode materials in the literature, among which the M-3–25 demonstrates significant advantages, which exhibit outstanding physicochemical structure, exceptional electrochemical characteristics, and promising applications of the investigated biochar electrode materials.

## 3. Materials and Methods

### 3.1. Synthesis of Nanoporous Activated Biochar

**Figure 8:**
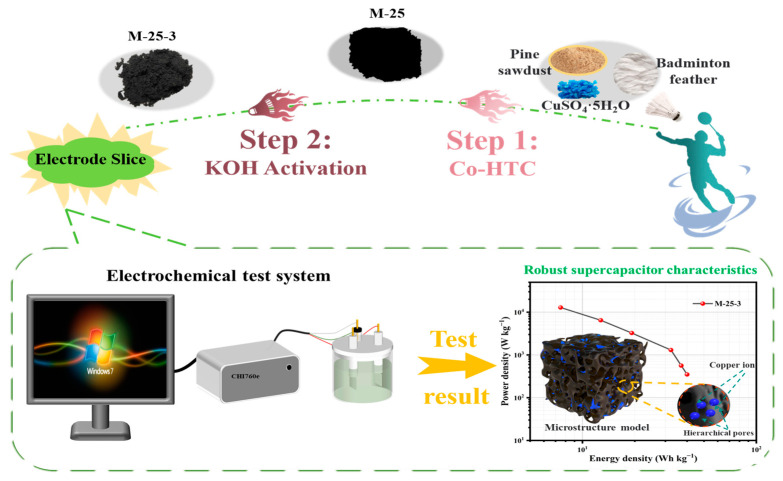
Schematic diagram of the preparation process of nanoporous activated biochar.

The raw materials, goose feathers and waste pine sawdust, were collected from discarded badminton and a furniture factory in China (Henan, Kaifeng), respectively. Figure 8 details the synthesis of nanoporous biochar samples. First, the waste pine sawdust and badminton feathers were dried in an oven at 80 °C for 12 h to a constant weight. The dried raw materials were then ground further in the crusher and passed through an 80-mesh sieve. To accurately adjust the effect of heavy metals throughout the experiment, the Cu-contaminated plants used in the reactions were synthesized using appropriate amounts of Cu and crushed pine sawdust according to reported methods [59,60,61]. The desired amounts of crushed pine sawdust and CuSO_4_·5H_2_O (Aladdin Industrial Corporation in Shanghai, China) were mixed in a beaker and stirred vigorously at 500 rpm for 6 h. The resulting mixtures (0, 25, 50 mg Cu/g biomass) and crushed badminton feathers were utilized together in the first step Co-HTC reaction. Then, 150 mL of ultrapure water and the desired amounts of Cu-contaminated plants (composed of 7.5 g of pine sawdust and the desired amount of Cu) and 7.5g badminton feathers were loaded into a 250 mL autoclave and heated up to 240 °C for 120 min. After the reactor had cooled down to room temperature, the HTC products were filtered with deionized water three times and poured into a culture dish, which was then dried to a constant weight at 80 °C for 12 h for further experiments. The dried solid HTC products were named PS-X and M-Y (pine sawdust and mixture), where X and Y represent the Cu content (mg) per gram in pine sawdust (X or Y = 0, 25, 50).

Using KOH (AR, Tianjin Kemio Chemical Reagent Co., Ltd., Tianjin, China) as the activator, the prepared hydro-char was mixed with KOH at weight ratios of 1: Z (Z = 2, 3, or 4) in a mortar and ground into the powder. The mixture was then placed in a nickel crucible and subjected to activation in a nitrogen atmosphere (25 mL min^−1^). The temperature was increased at a heating rate of 10 °C min^−1^ to 700 °C and held for 1 h. Then, the sample was naturally cooled to room temperature in a tubular furnace (CHY-1200, Henan Chengyi Equipment Technology Co., Ltd., Zhengzhou, China) under a nitrogen atmosphere. The activated sample was filtered three times with 0.1 M hydrochloric acid and deionized water three times until neutralized. The washed sample was dried in a culture dish at 80 °C to a constant weight. The activated samples were named PS-Z-Y and M-Z-Y, where Z represents the weight ratio of KOH (Z = 2, 3, or 4), and Y represents the Cu content (mg) per gram in pine sawdust (Y = 0, 25, 50). All experiments were conducted three times to ensure reproducibility.

### 3.2. Materials Characterization

The morphological structure of the samples was examined utilizing a field emission scanning electron microscope (SEM, ZEISS Gemini SEM 500, Oberkochen, Germany), and energy diffraction spectroscopy (EDS) was used to investigate the elemental composition. The specific surface area of the nanoporous biochar was analyzed employing the multipoint Brunauer–Emmett–Teller (BET, V-Sorb 2800P, Gold APP Instruments, Xi’an, China) method. The total pore volume (Vt) was calculated based on nitrogen adsorption measurements at a relative pressure of 0.99. The pore size distribution was determined using the Barrett–Joyner–Halenda method. The elemental contents of carbon (C), hydrogen (H), and nitrogen (N) in the samples were analyzed with an elemental analyzer (Elementar Vario EL cube, Frankfurt, Germany). The surface elemental composition and chemical groups of the nanoporous biochar samples were exhaustively characterized via X-ray photoelectron spectroscopy (XPS, AXIS SUPRA^+^, SHIMADZU, Buckinghamshire, UK). The crystal structure was systematically investigated using an X-ray diffractometer (D8-ADVANCE, Bruker, Dresden, Germany). Furthermore, Raman spectroscopic analysis was performed with a laser micro-Raman spectrometer (Renishaw, Wotton-under-Edge, UK).

### 3.3. Electrochemical Measurements

#### 3.3.1. Electrode Preparation

For the three-electrode system, 8 mg porous carbon simples, 1mg acetylene black, and 1 mg polytetrafluoroethylene (PTFE) binder (Aladdin Industrial Corporation, Shanghai, China) were mixed at a specific mass ratio of 8:1:1. The mixture was dispersed in anhydrous ethanol, serving as an emulsion-breaking agent, and thoroughly ground using a mortar and pestle to ensure homogeneity. The resulting composite, once uniformly ground, was then coated onto the square area (measuring 1.0 × 1.0 cm) of a nickel foam (AR, Tianjin Kemio Chemical Reagent Co., Ltd., Tianjin, China) sheet (measuring 1.0 × 2.0 cm). Finally, 1 × 1.5 cm nickel foam was used to cover the electrode material and the whole electrode slice pressed into a thin sheet under moderate pressure (10–20 MPa). For the two-electrode system, while maintaining the same method for the synthesis of the electrode slice, a polypropylene diaphragm was chosen for the construction of the symmetrical capacitors.

#### 3.3.2. Electrochemical Performance Measurements

The electrochemical evaluations encompassed cyclic voltammetry (CV), galvanostatic charge–discharge (GCD), and electrochemical impedance spectroscopy (EIS) reactions and cycling stability tests at room temperature, executed on an electrochemical workstation (CHI760E Chenhua, Shanghai, China). In the three-electrode setup, Pt and HgO/Hg served as the counter and reference electrodes, respectively, with 6 M KOH as the electrolyte. For the two-electrode configuration, a simulated double-layer capacitor was assembled with two identical 1.0 × 1.0 cm carbon electrodes separated by a polypropylene diaphragm, also utilizing 6 M KOH. EIS measurements were conducted at frequencies spanning from 0.1 Hz to 100 kHz, with an amplitude of 5 mV.

From the galvanostatic charge–discharge curve, the specific capacitance *C_sp_* (F g^−1^) Equation (1) of the three-electrode system can be calculated as follows:

(1) $$C_{\mathrm{sp}}=\frac{I\times ∆t}{m\times ∆V}$$

where $I\;\left(A\right)$ is the discharge current, ∆*t* (s) refers to the discharge time, *m* (g) is the mass of active material on the working electrode, and ∆*V* (V) refers to the potential change range during the discharge.

The formula for the specific capacitance of the two-electrode system can be calculated by Equation (2):

(2) $${C\prime }_{\mathrm{sp}}=\frac{I\times ∆t}{m\times ∆V}$$

where *C*′*_sp_* (F g^−1^) is the specific capacitance of a single electrode.

The calculation of energy density *E* (W h kg^−1^) and power density *P* (W kg^−1^) can be realized on the basis of Equations (3) and (4), respectively:

(3) $$E=\frac{C_{\mathrm{sp}}\times {\left(∆V\right)}^{2}}{8\times 3.6}$$

(4) $$P=\frac{E\times 3600}{∆t}$$

## 4. Conclusions

Herein, the present work demonstrated an easy and value-added utilization strategy for sports waste by co-HTC and pyrolysis activation. M-3-25 reached an ultrahigh specific capacitance of 587.14 F g^−1^ at 1 A g^−1^ and, after 10,000 cycles, its retention rate was high as 91.39% at a current density of 10 A g^−1^. In addition, the symmetrical supercapacitor M-3-25//M-3-25 also exhibited an excellent energy density of 42.16 W h kg^−1^ when the power density was 398.17 W kg^−1^ in the 6 M KOH electrolyte, which indicated an exceptional overall supercapacitor performance. Remarkably, compared with biochar from a single feedstock and typical biochar-based supercapacitors on the market (the energy/power density is usually 1–10 Wh/kg/<10,000 W/kg), when incorporating discarded badminton feathers as auxiliary materials into the hydrothermal carbonization process of pine sawdust, the obtained nanoporous biochar after pyrolysis activation displayed a superior porous structure and electrochemistry performance. Given the obstacles to managing huge amounts of sports waste, this work delivers a promising strategy for optimization of the resource utilization of sports waste, which offers an environmentally friendly and sustainable pathway that enables concurrent disposal of multiple solid waste biomass and synthesis of functional biochar-based electrode materials.

## Figures and Tables

**Table 1:** Porosity parameters, specific capacitance at 1 A g^−1^ of the samples.

Sample	S_BET_^a^ (m^2^ g^−1^)	V_total_^b^ (cm^3^ g^−1^)	V_micro_^c^ (cm^3^ g^−1^)	V_micro_/V_total_ (%)	V_meso_^d^ (cm^3^ g^−1^)	Capacity (F g^−1^)
PS-25	3.6	0.021	0.001	4.76	0.02	--
M-25	5.58	0.072	0.002	2.78	0.07	--
PS-3-0	1327.36	0.69	0.52	75.36	0.17	277.79
PS-3-25	1709.51	1.19	0.7	58.82	0.49	334.34
M-3-0	2309.43	1.20	0.95	79.17	0.25	438.49
M-2-25	1907.09	1.15	0.78	67.83	0.37	409.47
M-3-25	2501.08	1.40	1.02	72.86	0.38	587.14
M-4-25	2190.91	1.01	0.89	88.12	0.12	467.37
M-3-50	1555.15	0.83	0.64	77.11	0.19	688.91

^a^ S_BET_ is specific surface area by the BET method at P/P0 = 0.001–0.05. ^b^ V_total_ is total pore volume at P/P_0_ = 0.99. ^c^ V_micro_ is micropore volume and is evaluated by the t-plot method. ^d^ V_meso_ is mesopore volume and is evaluated by the BJH method.

**Table 2:** Elemental analysis of all samples.

**Sample**	**Yield (%)**	**C (%)**	**H (%)**	**N (%)**	**O (%) ^a^**	**O/C**	**H/C**
Pine Sawdust	--	45.81	6.48	1.96	45.75	0.75	1.70
Goose Feather	--	46.9	7.333	15.24	30.527	0.49	1.88
PS-0	53.32%	61.26	5.666	1.91	31.164	0.38	1.11
PS-25	48.35%	62.53	5.07	1.53	30.87	0.37	0.97
PS-50	47.27%	60.31	4.787	2	32.903	0.41	0.95
M-0	40.33%	58.56	4.51	6.555	30.375	0.39	0.92
M-25	41.26%	60.36	5.22	6.634	27.786	0.35	1.04
M-50	41.10%	53.15	5.855	4.74	36.255	0.51	1.32
PS-0-3	36.63%	86.56	3.193	1.95	8.297	0.07	0.44
PS-25-3	33.40%	73.7	3.158	1.78	21.362	0.22	0.51
PS-50-3	31.72%	66.06	2.637	1.88	29.423	0.33	0.48
M-3-0	36.70%	79.01	3.954	4.81	12.226	0.12	0.60
M-2-25	42.20%	58.46	4.346	3.59	33.604	0.43	0.89
M-3-25	30.86%	56.72	3.706	2.73	36.844	0.49	0.78
M-4-25	15.17%	64.35	2.323	1.61	31.717	0.37	0.43
M-3-50	25.47%	58.77	2.724	2.8	35.706	0.46	0.56

^a^ Calculated by difference.

**Table 3:** Comprehensive performance comparison of different electrode materials.

Electrode Material	SSA (m^2^ g^−1^)	Specific Capacitance (F g^−1^)	Current Density (A g^−1^)	Energy Density (Wh kg^−1^)	Power Density (W kg^−1^)	Cycle Stability (10 A g^−1^ %)	References
M-3-25	2501.08	587.14	1	42.16	398.17	91.39/10,000 cycles	This Work
Spirulina-based nanoporous biochar	2923.7	348	1	17.99	162.48	94.14/10,000 cycles	[22]
Zn-K-ATC	1975	384	1	--	--	72/ 10,000 cycles	[23]
zinc ion hybrid capacitor	686.29	154.64	0.1	--	--	--	[24]
N, O, S co-doped porous carbons	2864	270	1	11.84	8525	98/10,000 cycles	[35]

## Data Availability

Data are contained within the article and Supplementary Materials.

## Supplementary Materials

The following supporting information can be downloaded at: https://www.mdpi.com/article/10.3390/molecules30010026/s1, Figure S1: EDS mapping of M-3-25; Figure S2: SEM images of M-4-25; Figure S3: The equivalent circuit diagram of M-3-25
