# Supplementary material for: Co-Hydrothermal Carbonization of Goose Feather and Pine Sawdust: A Promising Strategy for Disposal of Sports Waste and the Robust Improvement of the Supercapacitor Characteristics of Pyrolytic Nanoporous Carbon
Source: Molecules. 2024 Dec 25;30(1):26. doi: 10.3390/molecules30010026 (PMC11722056; doi:10.3390/molecules30010026)
Supplement: Supplementary file 1 [file molecules-30-00026-s001.zip › molecules-3356117-supplementary.pdf]

Supplementary Materials

Co-Hydrothermal Carbonization of Goose Feather and Pine Sawdust: A Promising Strategy for Disposal of Sports Waste and the Robust Improvement of the Supercapacitor Characteristics of Pyrolytic Nanoporous Carbon

Tingyu Ma [1,2,†], Jieni Wang [1,3,†], Xiaobo Han [1,3], Chuanbing Zhang [3], Yahui Xu [3], Leichang Cao [1,3,*], Shuguang Zhao [3,*], Jinglai Zhang [1] and Shicheng Zhang [4]

[1] Henan Key Laboratory of Protection and Safety Energy Storage for Light Metal Materials, College of Chemistry and Molecular Sciences, Henan University, Kaifeng 475004, China; 17539120574@163.com (T.M.); jieniwang@henu.edu.cn (J.W.); 2138030016@henu.edu.cn (X.H.); zhangjinglai@henu.edu.cn (J.Z.)

[2] School of Physical Education and Sport, Henan University, Kaifeng 475004, China

[3] Huaxia Besince Environmental Technology Co., Ltd., Zhengzhou 450018, China; zhangchuanbing@besince.cn (C.Z.); xuyahui@besince.cn (Y.X.)

[4] Shanghai Key Laboratory of Atmospheric Particle Pollution and Prevention (LAP3), Department of Environmental Science and Engineering, Fudan University, Shanghai 200433, China; zhangsc@fudan.edu.cn

* Correspondence: clch666@henu.edu.cn (L.C.); zhaoshuguang@besince.cn (S.Z.)

† These authors contributed equally to this work.

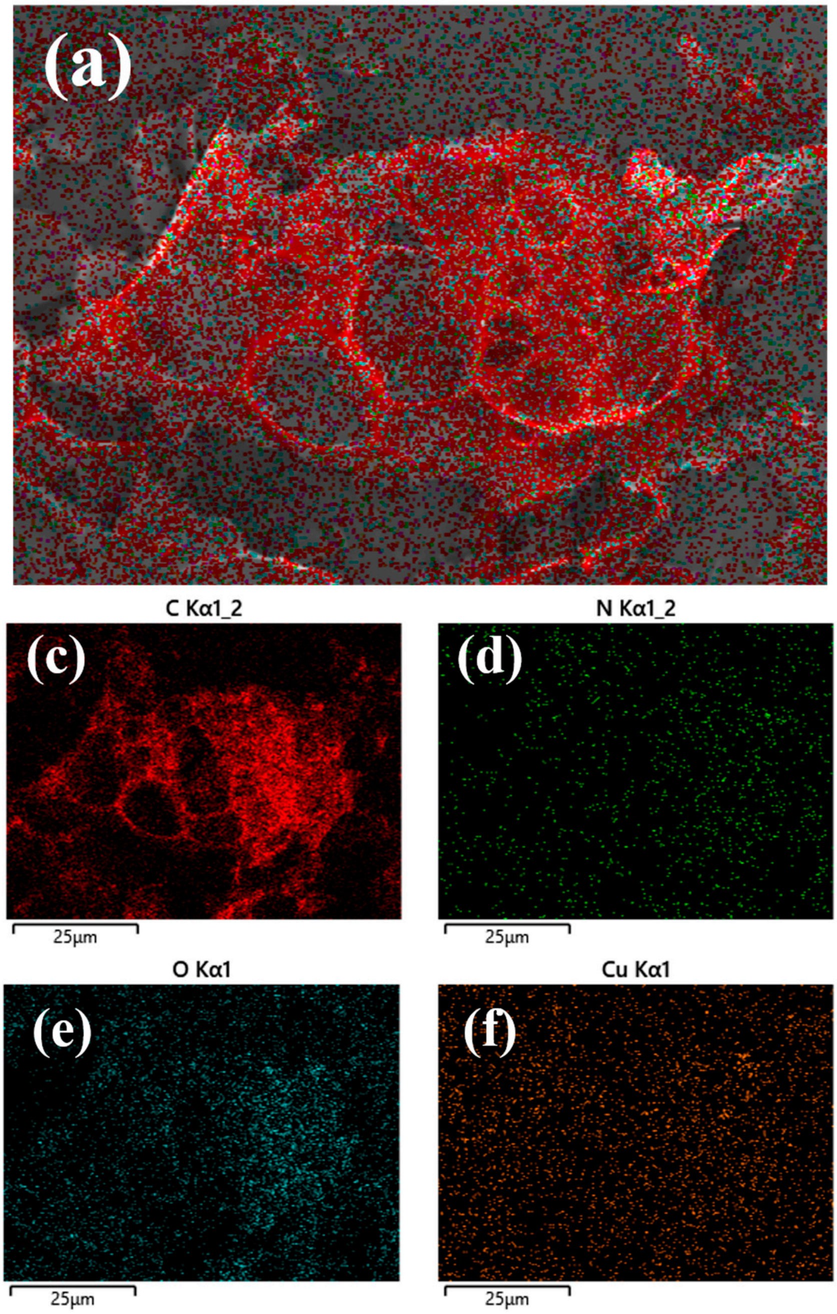

Figure S1. EDS mapping of M-3-25.

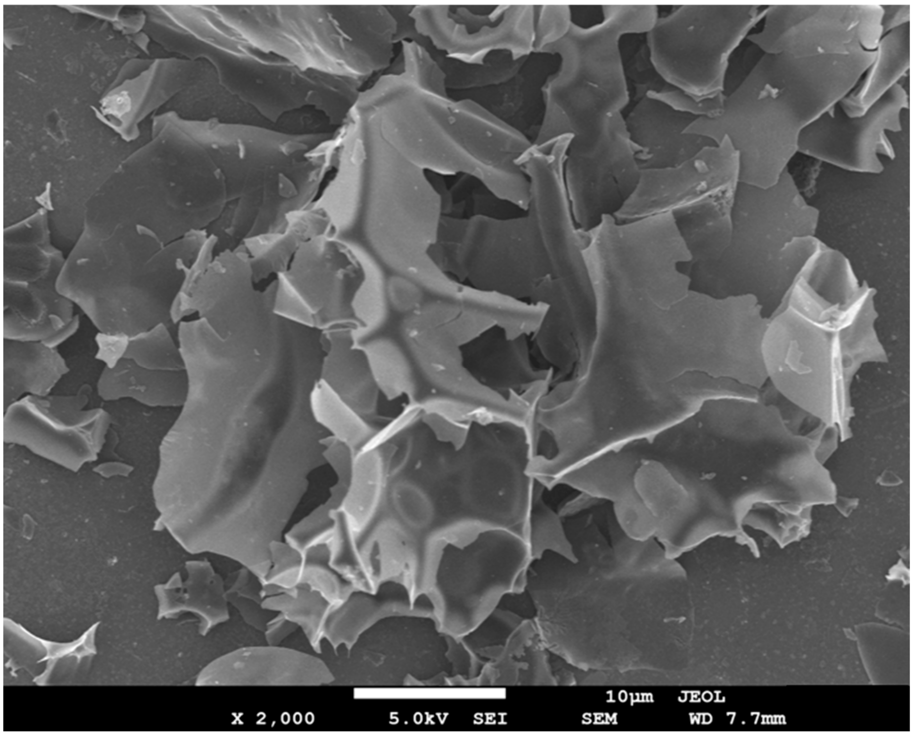

Figure S2. SEM images of M-4-25.

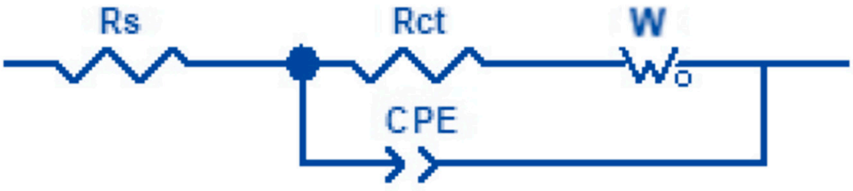

Figure S3. The equivalent circuit diagram of M-3-25.
